# Evaluation of Pharmaceutical Company Payments and Conflict of Interest Disclosures Among Oncology Clinical Practice Guideline Authors in Japan

**DOI:** 10.1001/jamanetworkopen.2019.2834

**Published:** 2019-04-26

**Authors:** Hiroaki Saito, Akihiko Ozaki, Toyoaki Sawano, Yuki Shimada, Tetsuya Tanimoto

**Affiliations:** 1 Department of Gastroenterology, Sendai Kousei Hospital, Sendai, Miyagi, Japan; 2 Medical Governance Research Institute, Shinagawa, Tokyo, Japan; 3 Department of Surgery, Minamisoma Municipal General Hospital, Minamisoma, Fukushima, Japan; 4 Department of Neurosurgery, Minamisoma Municipal General Hospital, Minamisoma, Fukushima, Japan

## Abstract

**Importance:**

Clinical practice guidelines (CPGs) are important in advancing the quality of medical care. Financial relationships between physicians and pharmaceutical companies may influence clinical practice. In accordance with the Japan Pharmaceutical Manufacturers Association guidelines for transparency, pharmaceutical company payments to physicians have been disclosed since 2013. The distribution of pharmaceutical company payments among CPG authors in Japan has not been studied.

**Objectives:**

To determine the characteristics and distribution of payments made by pharmaceutical companies to authors of oncologic CPGs in Japan and to assess the transparency of policies associated with conflict of interest (COI) disclosures in CPGs.

**Design, Setting, and Participants:**

This retrospective cross-sectional study of 326 authors from 6 prominent oncologic CPGs from Japan included annual payment data for 2016 from 78 pharmaceutical companies during varying times from January 1, 2016, through September 30, 2017.

**Main Outcomes and Measures:**

Amount and proportion of payments made by pharmaceutical companies to the authors; amount and proportion of payments made to the authors of each guideline; and information on policies for disclosing COIs in CPGs (Japanese yen were converted to US dollars based on the February 20, 2019 exchange rate of 110 yen per 1 US dollar).

**Results:**

Of 326 eligible authors, 255 (78.2%) received payments from pharmaceutical companies in 2016. The total number of payments was 3947, and the total amount was $3 444 193 (¥378 861 220), including $2 696 777 for speaking, $181 944 for writing, $554 381 for consulting, and $11 091 for unclear fees. The median payment amount was $3233 (interquartile range [IQR], $506-$10 873), and the mean (SD) payment amount was $10 565 ($20 059); 84 authors (25.8%) received more than $10 000. The largest proportions of CPG authors receiving at least 1 payment were those for gastric carcinoma (92%) and colorectal carcinoma (92%). The median payment was highest for authors of colorectal carcinoma guidelines ($7781; IQR, $2506-$18 633), whereas it was lowest for authors of pancreatic carcinoma guidelines ($2207; IQR, $304-$9240). Only breast carcinoma CPGs published the authors’ individual COI disclosure in an identifiable matter; guidelines for lung, colorectal, pancreatic, and hepatocellular carcinomas disclosed the financial relationships between the authors and companies anonymously; and the gastric carcinoma CPGs did not have a COI disclosure section.

**Conclusions and Relevance:**

Most oncologic CPG authors received payments from pharmaceutical companies, and COI disclosure methods appeared to be insufficient. Given the possibility of bias in guideline content if authors have any financial relationships with pharmaceutical companies, CPGs from Japan may require improved transparency.

## Key Points

**Question:**

What is the extent of payments from pharmaceutical companies to authors of oncologic clinical practice guidelines in Japan?

**Findings:**

In a cross-sectional study using databases from pharmaceutical companies, 255 of 326 authors (78.2%) of 6 prominent oncologic clinical guidelines received payments from pharmaceutical companies, with 25.8% receiving more than $10 000. Only guidelines for breast carcinoma published the authors’ individual conflict of interest disclosures in an identifiable matter.

**Meaning:**

These findings suggest the need for improved transparency in conflict of interest policy for authors of clinical practice guidelines in Japan.

## Teaser

This cross-sectional study evaluates the payment type and distribution from pharmaceutical companies, as well as policy transparency for conflict of interest disclosures among clinical practice guideline authors in Japan.

## Introduction

There is an increasing focus on transparency in the financial relationships between the pharmaceutical industry and physicians worldwide because such financial relationships can bias physicians’ decision making^1,2,3,4,5^ and a large amount of payments has been made for promotional purposes. To enable a fair evaluation of the standpoints among physicians engaged in academic work, individual physicians should fully and carefully disclose corporate financial conflicts of interest (COIs). In the United States,^6^ Australia,^7^ most of the European countries,^8^ and Japan,^9^ pharmaceutical companies have been required to publicly report the payments they provide to physicians. In the United States, payment data from pharmaceutical and medical device industries have been available since 2013 through the Open Payments program; the United Kingdom has required these data since 2016. Previous studies have suggested that board specialists, medical journal editors,^10^ executive board members of professional medical associations, and clinical practice guideline (CPG) authors^11,12,13^ are critical targets for pharmaceutical payments.

Among various types of representatives in medical fields, CPG authors exert some of the largest influences on clinical practice^14^ because they present recommendations for drugs and other treatment modalities for specific disorders. Thus, CPG authors may be prime targets for pharmaceutical company payments used for promotional purposes despite self-regulation and other policies; this could be particularly true in oncology. With aging populations, pollution, and poor or excessive nutrition affecting populations, cancer has become an increasing problem. With the increasing rates of cancer, through great efforts, methods for prevention and treatment of certain cancers have been investigated and successfully developed. To develop cancer treatment drugs efficiently, the pharmaceutical industry has adopted a new business model—the discovery and development of anticancer agents that can be sold at high prices. For example, a single administration of tisagenlecleucel, a recently approved chimeric, antigen-receptor T-cell immunotherapy treatment manufactured by Novartis Pharma reportedly costs US $475 000.^15,16^

In Japan, the third largest pharmaceutical market in terms of annual pharmaceutical sales in 2016, sales of anticancer drugs exceeded 1.1 trillion billion yen (US $10 billion) in 2017 and are estimated to reach 1.4 trillion yen (US $13 billion) by 2025.^17^ Thus, oncology is a strategically important market for pharmaceutical companies in Japan, and evaluating the financial relationships among CPG authors and pharmaceutical companies is important. The Japan Pharmaceutical Manufacturers Association formulated guidelines about transparency of pharmaceutical payments to physicians in 2012.^18^ In accordance with these guidelines, each pharmaceutical company affiliated with the Japan Pharmaceutical Manufacturers Association has publicly disclosed their payment data since 2013. These data enabled us to perform a comprehensive assessment of industry payments to CPG authors in Japan. However, because of the inconsistency of the platforms for payment disclosure between companies, few studies have assessed the financial relationships between physicians and pharmaceutical companies in Japan.^19,20^ Recently, a study^21^ reported that a large number of executive board members of notable professional medical associations in Japan received payments from pharmaceutical companies that totaled $6 468 585 in 2016.

The aims of the present study were to determine the characteristics and distributions of payments made to authors of CPGs in Japan and to assess the transparency of policies for COI disclosures in CPGs.

## Methods

### Study Population

We analyzed pharmaceutical payments made to the authors of the 6 oncology CPGs with the greatest influence on clinical practice. According to the National Cancer Center Japan, 372 986 people died of cancer in Japan in 2016.^22^ For males, the top 5 types of cancer death were lung, gastric, colorectal, hepatocellular, and pancreatic cancers, and for females, colorectal, lung, pancreatic, gastric, and breast cancers. We reviewed the prominent CPGs associated with these cancers in Japan and chose 6 CPGs published from October 20, 2016, through May 16, 2018. The CPG authors were chosen at the discretion of related medical societies. Table 1 shows characteristics of the 6 oncology CPGs.^23,24,25,26,27,28^ We identified and included all the CPG authors. We collected information about their medical specialties, affiliations, and positions at their affiliations by reviewing the CPGs or the webpage of the authors’ affiliations. In addition, details of COI policies in the CPG were collected; these details included whether the CPGs have COI sections, whether individual COIs were disclosed in the relevant sections, and whether details of COI disclosure were publicly available. We collected details about individual author COIs if available. We verified whether the value of the payment received based on our database fell within the guideline’s criterion of COI disclosure. The study followed the Strengthening the Reporting of Observational Studies in Epidemiology (STROBE) reporting guideline. Institutional review board approval was obtained from the Committee on the Medical Governance Research Institute, Minato-ku, Tokyo, Japan. Informed consent from the authors of CPGs was not obtained because the payment data collected and analyzed were provided publicly from each pharmaceutical company.

**Table 1.** Characteristics of Clinical Practice Guidelines

Guidelines	Editorial Associations	Date of Publication	Time Frame for Reporting COIs^a^
Guidelines for gastric cancer treatment^23^	Japanese Gastric Cancer Association	January 31, 2018	None^b^
Guidelines for the treatment of colorectal cancer^24^	Japanese Society for Cancer of the Colon and Rectum	November 11, 2016	None^b^
Hepatocellular carcinoma guidelines^25^	The Japan Society of Hepatology	October 20, 2017	January 1, 2014, to December 31, 2016
Guidelines for diagnosis and treatment of lung cancer^26^	The Japan Lung Cancer Society	December 19, 2017	None^b^
Guidelines for diagnosis and treatment of pancreas cancer^27^	Japan Pancreas Society	October 20, 2016	January 1, 2013, to December 31, 2015
Clinical practice guidelines for breast cancer^28^	Japanese Breast Cancer Society	May 16, 2018	January 1, 2015, to December 31, 2017

Abbreviation: COIs, conflicts of interest.

^a^ Time frame for reporting COIs was described in each guideline.

^b^ Time frame for reporting COIs was unclear both in the guidelines and on the webpages of their editorial associations.

### Sources of Payment Data

We collected payment data from the 2016 fiscal year that were published by all 71 companies that belonged to the Japan Pharmaceutical Manufacturers Association and 7 other pharmaceutical companies adhering to the Japan Pharmaceutical Manufacturers Association transparency guidelines. For most of the eligible companies, the 2016 data were the most recent payment data and previous data were not available. The companies included in this study and the starting and ending dates of their payment data are listed in eTable 1 in the Supplement. Data included physicians’ names, their main institution, the amount of payments received, the form of payments, and the total records of payments. The form of payment was categorized into 3 types: speaking, writing, and consulting fees.

Because no unified and ready-made database encompassing all the companies was available, we obtained each company’s data individually and organized the data into a unified database through the following steps. First, because no data were published as a spreadsheet, data with character codes were converted into a spreadsheet format. Second, data with no character code were converted into text files using an optical character reader (Yomitori kakumei, version 15; Panasonic Solution Technologies Company, Ltd). Third, when disclosed data were protected against facsimile or reproduction, we used FullShot10 software (Inbit Inc) to scan photos of the data and converted the data into text files. Fourth, we confirmed the accuracy of the organized data by comparing them with the original data and finalized the payment database for the 2016 fiscal year.

### Statistical Analysis

To determine characteristics and distributions of the pharmaceutical payments, we conducted descriptive analyses of the global payment data. We converted Japanese yen to US dollars using the February 20, 2019, exchange rate of 110 yen per 1 US dollar. We calculated the proportion of authors who received at least 1 payment and the mean and median value of payments among all authors of each guideline. When calculating mean and median payments, we included the zero values. To elucidate the existing policies on COI disclosure, we descriptively analyzed the COI policies in the CPGs. When possible, we elucidated the accuracy of the COI disclosure among the authors, on an individual basis, by comparing their disclosure with the payment data. In each CPG, we assessed the availability of the time frame for disclosing COIs among the authors.

All analyses were conducted using Microsoft Excel, version 14.5 (Microsoft Corp) and Stata, version 14.2 (StataCorp).

## Results

Of 6 oncologic guidelines reviewed, 326 authors were abstracted as follows: gastric carcinoma guidelines (n = 26), colorectal carcinoma guidelines (n = 25), lung carcinoma guidelines (n = 91), hepatocellular carcinoma guidelines (n = 68), breast carcinoma guidelines (n = 72), and pancreatic carcinoma guidelines (n = 50). One anonymous author of the pancreatic carcinoma guidelines, as the representative of the patients, was excluded. Among the others, 6 authors worked for more than 1 guideline: colorectal carcinoma and pancreatic carcinoma (n = 2); hepatocellular carcinoma and pancreatic carcinoma (n = 1); gastric carcinoma and colorectal carcinoma (n = 1); hepatocellular carcinoma and colorectal carcinoma (n = 1); and lung carcinoma and pancreatic carcinoma (n = 1).

Of 326 authors contributing to CPG development, 255 (78.2%) received at least 1 payment; 84 (25.8%) accepted more than $10 000; 17 (5.2%) accepted more than $50 000; and 3 (0.9%) accepted more than $100 000. There were 3947 total payments, and the total amount was $3 444 193 (¥378 861 220), including $2 696 777 (78.3%) for speaking, $181 944 (5.3%) for writing, and $554 381 (16.1%) for consulting. The rest of the payment accounting for $11 091 (0.3%) comprised unclear fees. The median payment amount was $3233 (interquartile range [IQR], $506-$10 873), and the mean (SD) payment amount was $10 565 ($20 059).

Table 2 shows details of payments among each CPG author. The proportion of CPG authors receiving at least 1 payment was largest for gastric (24 of 26; 92%) and colorectal carcinomas (23 of 25; 92%), followed by lung (70 of 91; 77%), pancreatic (38 of 50; 76%), breast (54 of 72; 75%), and hepatocellular carcinomas (50 of 68; 74%). Furthermore, 11 of 26 (42%) authors of gastric carcinoma CPGs and 11 of 25 (44%) authors of colorectal carcinoma CPGs received $10 000 or more, whereas less than 30% of the authors received this payment amount for other CPGs. The Figure presents a distribution of the monetary payment value for each CPG. The median value of the payment was largest for the colorectal carcinoma CPG ($7781; IQR, $2506-$18 633), followed by gastric ($6440; IQR, $3971-$25 192), hepatocellular ($3057; IQR, $0-$7550), lung ($2560; IQR, $312-$11 584), breast ($2538; IQR, $101-$7546), and pancreatic ($2207; IQR, $304-$9240) carcinoma CPGs.

**Table 2.** Payments to Authors by Type of Oncology

Topics of Guideline	Total Payment^a^		Authors Receiving Payment, No. (%)		
	Payment Count, No.	Payment Amount, $	Any	≥$10 000	≥$50 000
Gastric carcinoma (n = 26)	526	443 372	24 (92)	11 (42)	1 (4)
Colorectal carcinoma (n = 25)	526	406 414	23 (92)	11 (44)	2 (1)
Lung carcinoma (n = 91)	1312	1 157 327	70 (77)	25 (27)	8 (9)
Hepatocellular carcinoma (n = 68)	701	711 139	50 (74)	14 (21)	5 (7)
Breast carcinoma (n = 72)	679	524 652	54 (75)	14 (19)	1 (1)
Pancreatic carcinoma (n = 50)	378	314 651	38 (76)	11 (22)	1 (2)
Total (N = 326)^b^	3947	3 444 193	255 (78)	84 (26)	17 (5)

^a^ The value of payment is described based on the exchange rate on February 20, 2019: 110 yen per 1 US dollar.

^b^ Columns may not add to the total of all clinical guidelines because the data were adjusted for 6 authors who worked for 2 guidelines.

**Figure.**
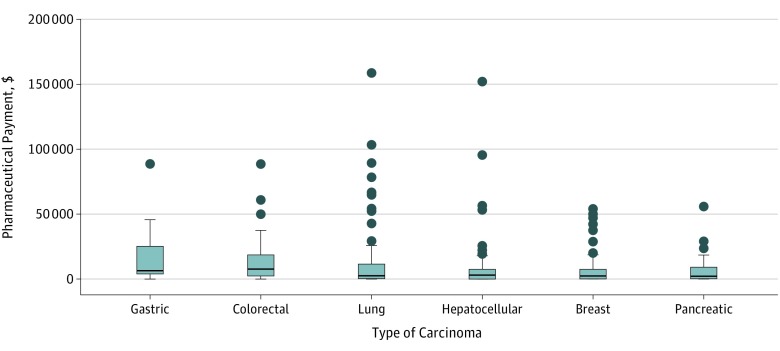
Distribution of the Value of Payments Received by Authors of 6 Oncologic Clinical Guidelines Box plot denotes the medians (inner horizontal lines) and interquartile ranges (outer horizontal lines). Bars outside the box represents the upper or lower adjacent value; circles represent outside values. Payment values were converted to US dollars using the February 20, 2019, exchange rate of 110 yen per 1 US dollar.

A chairperson of lung carcinoma ($158 217) and liver carcinoma ($152 156) CPGs received the largest value payments. All other chairpersons received at least 1 payment for gastric ($16 194), breast ($47 147), colorectal ($17 953), and pancreatic ($506) carcinoma CPGs.

When we examined the COI disclosure policy for each CPG, only the breast carcinoma CPG published the authors’ individual COI disclosures in an identifiable matter. The lung, colorectal, pancreatic, and hepatocellular carcinoma guidelines disclosed the financial relationships between the authors and companies anonymously, and the CPG for gastric carcinoma did not have a section for COI disclosure. Each CPG set the criteria with which the authors disclosed their financial relationships with the industry to the administrative office of the CPG (eTable 2 in the Supplement). Overall, the minimum monetary value of the financial relationships set by each CPG was uniform among CPGs.

Further analysis was conducted for the breast cancer CPG; 54 authors of the breast cancer CPG had pharmaceutical payments in our database. Of the 54 authors, 17 authors met the criteria set by the CPG to declare a COI relationship according to the database. However, 1 author did not have a COI statement for pharmaceutical relationships in the guideline, although the other 16 authors did.

Table 3 shows the list of the pharmaceutical companies that provided the top 5 largest monetary values of payments to the authors of each CPG. All of the listed pharmaceutical companies manufactured products for each type of cancer.

**Table 3.** Companies Providing the Most Payments per Guideline

Guideline, Company	Authors, No. (%)	Value, $^a^	Count, No.
Gastric (n = 26)			
Taiho Pharmaceutical Co Ltd	17 (65)	94 065	97
Chugai Pharmaceutical Co Ltd	18 (69)	75 353	93
Eli Lilly Japan KK	15 (58)	66 249	81
Takeda Pharmaceutical Company Ltd	7 (27)	30 895	40
Yakult Honsha Co Ltd	11 (42)	23 555	27
Colorectal (n = 25)			
Chugai Pharmaceutical Co Ltd	15 (60)	87 433	120
Taiho Pharmaceutical Co Ltd	18 (72)	64 954	82
Takeda Pharmaceutical Company Ltd	12 (48)	47 127	58
Eli Lilly Japan KK	7 (28)	46 277	69
Merck Serono Co Ltd	13 (52)	41 880	46
Lung cancer (n = 91)			
AstraZeneca	37 (41)	190 848	219
Chugai Pharmaceutical Co Ltd	41 (45)	186 052	220
Ono Pharmaceutical Co Ltd	33 (36)	145 895	159
Eli Lilly Japan KK	28 (31)	136 125	160
Nippon Boehringer Ingelheim Co Ltd	36 (40)	130 400	135
Liver (n = 68)			
AbbVie GK	15 (22)	93 830	78
Bayer Yakuhin Ltd	27 (40)	87 855	84
Bristol-Myers Squibb Co	12 (18)	55 400	48
MSD KK	9 (13)	47 212	35
Eisai Co Ltd	22 (32)	45 164	52
Breast (n = 72)			
Chugai Pharmaceutical Co Ltd	34 (47)	126 822	174
Eisai Co Ltd	25 (35)	63 476	80
Novartis Pharma KK	22 (31)	63 247	82
Kyowa Hakko Kirin Co Ltd	23 (32)	48 177	58
AstraZeneca	22 (31)	44 905	59
Pancreas (n = 50)			
Taiho Pharmaceutical Co Ltd	26 (52)	89 501	114
EA Pharma Co Ltd^b^	14 (28)	28 874	34
Daiichi Sankyo Co Ltd	13 (26)	22 299	29
Eisai Co Ltd	8 (16)	18 022	11
MSD KK	9 (18)	16 497	14

^a^ The value of payment is described based on the exchange rate on February 20, 2019: 110 yen per 1 US dollar.

^b^ EA Pharma Co Ltd was established on April 1, 2016.

## Discussion

Although there have been studies analyzing industrial payments among journal editors, clinical researchers, and CPGs in several countries, few investigations have assessed the financial relationships between physicians and pharmaceutical companies in Japan. In the analysis of 326 authors of oncologic CPGs in Japan, we revealed, for the first time to our knowledge, that 78.2% of the authors of CPGs received pharmaceutical payments. We also found that there were differences in the policies for COI disclosures among guidelines.

In 2016, Mitchell et al^12^ reported that 84% of National Comprehensive Cancer Guideline authors received a mean of $10 011 in general payments. Furthermore, in 2018, Khan et al^29^ reported that 56.9% of authors of CPGs including high-revenue medication had financial COIs. Consistent with these studies, authors of most of the 6 prominent oncologic CPGs in Japan received pharmaceutical payments, and the mean monetary value of the payments was large ($10 565). These findings confirm our hypothesis that there have been strong financial relationships between oncologic CPG authors and pharmaceutical companies. Another plausible explanation of the results was our selection of the CPGs of the 6 cancer types associated with the largest number of deaths in Japan. The number of patients could be positively associated with the market size and the priority of promotional activities because a high-level recommendation in each CPG can significantly affect the drug sales under universal health coverage for approved drugs in Japan.

Pharmaceutical companies may provide more occasions for speaking, writing, and providing payments to those with leading roles and a large influence among the authors of each CPG. Of note, every chairperson received some payment, with 2 chairpersons (of hepatocellular carcinoma and lung carcinoma CPGs) receiving the largest payments. Given their roles, such payment may influence the overall decision-making process of a physician in a guideline committee. A large disparity was observed in the amount of payments received by 255 CPG authors; only 84 of 326 authors (26%) received more than $10 000. This finding is consistent with previous findings.^12,13,14^ As for the differences in median value among guidelines, the number of authors could be a factor associated with the mean monetary value of the payment. For instance, the rate of authors receiving more than $10 000 in gastric carcinoma CPGs and colorectal carcinoma CPGs was high compared with this rate for the other CPGs. Because these 2 guidelines had a small number of authors, each author may have had more influence on the content of the guidelines and may have drawn larger payments for each author compared with other guidelines that had more members.

Authors of lung carcinoma CPGs received the largest payments in total compared with the authors of the other CPGs. This finding may reflect the current competitive situation in the lung carcinoma drug market in Japan; there are multiple novel and expensive oncology drugs in Japan, particularly for non–small-cell lung cancer, such as nivolumab, pembrolizumab, alectinib, osimertinib, ramucirumab, and afatinib. This field may be a critical target for advertisement with numerous rival companies, and each company may have allocated large-value payments to gain superiority compared with other companies, leading to the largest payments for authors of the lung cancer CPGs.

The current platform for COI disclosure in oncologic CPGs did not properly reveal the financial relationships of authors with industrial companies. The financial relationships of the CPG authors should be available to the general public.^14,30^ There is an opinion that authors that have a financial relationship with pharmaceutical companies need not necessarily be excluded and that it depends on the levels of conflicts.^14^ However, there is also a further demand to more rigorously control COI policies in CPGs: those authors with any COIs should not permitted to be included as CPG authors.^31^ All relevant stakeholders involved in the selection of CPG authors should recognize the importance of completely and correctly disclosing the financial COIs of each author. As for oncologic guidelines and other CPGs in Japan, the authors should declare industrial payments regardless of the amount, and the guidelines committee should provide the opportunity to declare them publicly. An external public review may be necessary to manage COIs actively and correctly. We believe that authors with high-level conflicts should be excluded from CPG committees; therefore, the definition of the high level of COI may need further discussion.

### Limitations

There are some limitations in this study. First, there might be measurement errors in the database. Although the accuracy of the data was carefully and repeatedly reviewed, the database might include human-induced errors because the data were manually entered. The format of payment data and measures of its disclosure significantly differed among pharmaceutical companies. It was uncommon among pharmaceutical companies to disclose the data in a readily available format, such as a spreadsheet. We recommend more organized and user-friendly information disclosure regarding payment data. Second, the present research analyzed only pharmaceutical companies with limited payment types. Without taking payment from device companies and other types of payment, such as royalties, into account, the financial relationship of CPG authors with industrial companies might have been underestimated. Further extensive research is required.

## Conclusions

In Japan, 78% of authors of 6 prominent oncologic CPGs received payments from pharmaceutical companies, but the methods of COI disclosure appeared to be insufficient. Given the possibility of bias in CPG content if authors have any financial relationships with pharmaceutical companies, improved transparency may be required.

## Notes

Supplement. **eTable 1.** 78 Pharmaceutical Companies and Their Period of Aggregating the Payments **eTable 2.** Guideline COI Criteria for Disclosure: Annual Payment
